# Effect of sarcopenia on short- and long-term outcomes in patients with gastric neuroendocrine neoplasms after radical gastrectomy: results from a large, two-institution series

**DOI:** 10.1186/s12885-020-07506-9

**Published:** 2020-10-15

**Authors:** Jia-bin Wang, Zhen Xue, Jun Lu, Qing-liang He, Zhi-fang Zheng, Bin-bin Xu, Jian-wei Xie, Ping Li, Yu Xu, Jian-xian Lin, Qi-yue Chen, Long-long Cao, Mi Lin, Ru-hong Tu, Ze-ning Huang, Ju-li Lin, Chang-ming Huang, Chao-hui Zheng

**Affiliations:** 1 Department of Gastric Surgery, Fujian Medical University Union Hospital, No.29 Xinquan Road, Fuzhou, 350001 Fujian Province China; 2 Department of General Surgery, Fujian Medical University Union Hospital, Fuzhou, China; 3 Key Laboratory of Ministry of Education of Gastrointestinal Cancer, Fujian Medical University, Fuzhou, Fujian Province China; 4 Department of gastrointestinal surgery, the first Affiliated Hospital of Fujian Medical University, Fuzhou, Fujian Province China; 5 Department of Pathology, the School of Basic Medical Sciences, Fujian Medical University, Fuzhou, China

**Keywords:** Gastric neuroendocrine neoplasms, Sarcopenia, Overall survival, Risk factors

## Abstract

**Background:**

The relationship between sarcopenia and the prognoses of patients with gastric neuroendocrine neoplasms (g-NENs) is unclear. This study was designed to explore the effects of sarcopenia on short-term and long-term outcomes of patients with g-NENs after radical gastrectomy.

**Methods:**

This study retrospectively collected data from 138 patients with g-NENs after radical gastrectomy. The skeletal muscle index (SMI) diagnostic threshold for sarcopenia was determined using X-tile software. Cox regression analyses were performed to determine the independent risk factors for 3-year overall survival (OS) and 3-year recurrence-free survival (RFS).

**Results:**

In this study, 59 patients (42.8%) were diagnosed with sarcopenia. Among patients in the sarcopenia group and nonsarcopenia group, the incidences of total postoperative complications were 33.9 and 30.4%, incidences of serious postoperative complications were 0 and 3.7%, incidences of postoperative surgical complications were 13.6 and 15.2%, and incidences of postoperative systemic complications were 20.3 and 15.2%, respectively (all *p* > 0.05). The 3-year OS and RFS rates were significantly worse in the sarcopenia group than in the nonsarcopenia group (OS: 42.37% vs 65.82%, *p* = 0.004; RFS: 52.54% vs 68.35%, *p* = 0.036). The multivariate analysis revealed a relation between sarcopenia and the long-term prognoses of patients with g-NENs. A stratified analysis based on the pathological type revealed that the Kaplan-Meier curve was only significantly different in patients with gastric mixed adenoneuroendocrine carcinoma (gMANEC) (OS: 40.00% vs 71.79%, *p* = 0.007; RFS: 51.43% vs 74.36%, *p* = 0.026); furthermore, the multivariate analysis identified sarcopenia as an independent risk factor for patients with gMANEC (*p* < 0.05).

**Conclusions:**

Sarcopenia is not related to the short-term prognoses of patients with g-NENs. Sarcopenia is an independent risk factor for patients with gMANEC after radical surgery.

## Synopsis

This study was designed to explore the effects of sarcopenia on the short-term and long-term outcomes of patients with g-NENs after radical gastrectomy by using data from two independent large-volume institutions.

## Background

Gastric neuroendocrine neoplasms (g-NENs) are a class of tumors with significant heterogeneity that account for approximately 4% of all neuroendocrine tumors [1], and their incidence is gradually increasing [2, 3]. g-NENs include three categories: gastric neuroendocrine tumor (gNET), gastric neuroendocrine carcinoma (gNEC) and gastric mixed adenoneuroendocrine carcinoma (gMANEC) [4]. Surgery is the main treatment for gNET, gNEC, gMANEC [5]. Because of its different clinicopathological features, the understanding and the prognostic factors of g-NENs are still rarely studied [6–9]. Therefore, studies exploring the factors influencing the short-term and long-term outcomes of patients with g-NENs after radical surgery are important to improve the prognosis of these patients.

In recent years, sarcopenia has been reported to be closely related to the prognosis of patients with gastric cancer, liver cancer, colorectal cancer, and other malignant tumors [10–16]. However, no studies have reported the effect of sarcopenia on the short-term and long-term postoperative outcomes of patients with g-NENs.

This study retrospectively analyzed the clinicopathological data from 138 patients with g-NENs treated at two institutions, with the aim of exploring the effect of sarcopenia on the short-term and long-term outcomes of patients with g-NENs after radical gastrectomy.

## Methods

### Patient selection

The clinicopathological data from patients diagnosed with g-NENs at the Fujian Medical University Union Hospital (FMUUH) and the First Affiliated Hospital of Fujian Medical University (FMUFAH) from December 2009 to December 2015 were retrospectively analyzed. The inclusion criteria were as follows: (1) patients who were diagnosed with g-NENs by pathology; (2) patients without distant metastasis, as assessed by a preoperative examination; and (3) patients who underwent R0 excision. The following exclusion criteria were used: (1) distant metastasis was identified preoperatively and intraoperatively; (2) patients received neoadjuvant chemotherapy or radiotherapy before surgery; and (3) basic clinical data and computed tomography (CT) images were incomplete. One hundred thirty-eight patients with g-NENs were finally included in this study (111 patients at FMUUH and 27 patients at FMUFAH, Supplementary Table 1). The tumor size, location, T stage and N stage were comprehensively determined by two attending physicians according to the findings of gastroscopy, abdominal CT and other auxiliary examinations performed preoperatively. The type of surgical resection performed was determined by the location of the tumor. Lymph node dissection was performed according to the Japanese gastric cancer treatment guidelines (13th edition) [17]. For patients with gNET, somatostain was recommended. For patients with stage II or higher gNEC or gMANEC, fluorine-based postoperative adjuvant chemotherapy was recommended [18]. The study was approved by the Ethics Committees of FMUUH and FMUFAH.

### Diagnosis and classification of g-NENs

According to the 2010 WHO classification of tumors of the digestive system [4], g-NENs were classified as gNET, including NET1 and NET2 grades; gNEC, including large-cell carcinomas and small-cell carcinomas; and gMANEC. Neuroendocrine cells were confirmed, diagnosed and classified based on the microscopic histomorphological features and immunohistochemical staining for neuroendocrine tumor-related biomarkers (such as CgA, CD56 and Syn). The pathological findings were confirmed by two experienced pathologists.

### Analysis of CT images

A preoperative abdominal CT scan within 1 month of surgery was considered to accurately reflect the patient’s muscle status. A researcher who was blinded to the outcome measured the skeletal muscle cross-sectional area (cm^2^) at the level of the third lumbar vertebra (L3) by using Osirix 3.3 software (32-bit; http://www.osirix-viewer.com) [19]. The researcher was trained to accurately identify lumbar vertebrae and muscles (Supplementary Fig. 1). The average surface area (cm^2^) of two consecutive slices was used for analysis. If necessary, the area of the selected area was manually adjusted to accurately calculate the area. The tissue discrimination threshold of skeletal muscle is − 29 to + 150 Hounsfield units (HUs) [20]. The muscle area (cm^2^) was standardized to the height (m^2^) to obtain the L3 skeletal muscle index (SMI) (cm^2^/m^2^) [21].

### Optimal SMI cutoff value and definition of sarcopenia

Separate X-tile plots were constructed for men and women. For the men, when the SMI value was 44.3 cm^2^/m^2^, the maximum chi-square log-rank value of 4.2611 was achieved. Therefore, a SMI ≤ 44.3 cm^2^/m^2^ was defined as sarcopenia, and a SMI > 44.3 cm^2^/m^2^ was defined as nonsarcopenia (*p* = 0.038) (Supplementary Fig. 2).

For the women, a SMI ≤ 32.4 cm^2^/m^2^ was defined as sarcopenia in the same manner (χ^2^ = 1.0039, *p* = 0.214) (Supplementary Fig. 2).

### Variables and definitions

Overall survival (OS) was defined as the time from surgery to the last follow-up, death, or the last record in the follow-up database (such as loss of follow-up or death from other diseases). Recurrence-free survival (RFS) was defined as the time from surgery to the initial recurrence. Postoperative complications were classified according to the Clavien-Dindo criteria [22]. Total postoperative complications were defined as Clavien-Dindo grade 2 and higher. Severe complications were defined as Clavien-Dindo grade 3 and higher [11]. Postoperative surgical complications were defined as complications related to the surgical procedure. Systemic complications were defined as complications that were not directly related to the surgical field or the incision. For Ki-67, 60% positive was considered the cut-off point. The ASA physical status classification system was used in this research [18, 23]. ASA I, patient is healthy with no systemic disease; ASA II, patient has mild systemic disease; ASA III, patient has severe systemic disease or multiple diseases affecting different organ systems; ASA IV, patient has severe systemic disease that is a constant threat to life; ASA V, patient is moribund and not expected to survive without the operation; and ASA VI, brain dead patient whose organs are being removed for donation [24, 25].

### Follow-up

The median follow-up time was 36 months (range: 1–102 months). Physical and laboratory examinations were performed regularly after surgery, once every 3 months for 2 years, every 6 months for the next 3 years, and once a year after 5 years. In addition, imaging examinations, including chest radiographs, abdominal and pelvic CTs, and endoscopy, were performed at least once a year. If necessary, additional MRI or PET studies were performed to determine whether recurrence was present.

### Statistical analysis

All data were statistically analyzed using SPSS 22.0 software. Continuous variables are reported as the means ± SD or medians (interquartile ranges). X-tile plots were used as a new bioinformatics tool for biomarker assessments and outcome-based cutoff point optimization [10, 26]. Categorical and continuous variables were compared using a χ^2^ test or Fisher’s exact test and a t-test, respectively. The OS and RFS rates were calculated by the Kaplan-Meier method, and the differences were assessed with log-rank tests. The Cox proportional hazards regression model was used to analyze the independent prognostic factors for 3-year OS and RFS rates. *P* values less than 0.05 were considered statistically significant.

## Results

### Clinicopathological characteristics

Among the 138 patients, 59 patients (42.8%) were included in the sarcopenia group and 79 patients (57.2%) were included in the nonsarcopenia group. A total of 12 gNET patients, 52 gNEC patients, and 74 gMANEC patients were included in this study. Of gNET patients, 6 patients were type 1, 5 patients were type 2, and 1 patient was type 3. The comparison of clinical data between the two groups showed a higher incidence of sarcopenia in the subgroups of male patients, aged 65 years, with a BMI of < 25 and a tumor larger than 50 mm (all *p* < 0.05). However, no significant differences in the other variables were observed between the two groups (all *p* > 0.05) (Table 1).

**Table 1:** Clinicopathological characteristics

Variable	All (*n* = 138)	Low (*n* = 59)	High (*n* = 79)	P
Gender				0.014
Male	105	51	54	
Female	33	8	25	
Age(years)				0.004
< 65	80	26	54	
≥ 65	58	33	25	
BMI(kg/m2)				0.007
< 25	115	55	60	
≥ 25	23	4	19	
ASA				0.664
1	69	28	41	
2	54	23	31	
3	15	8	7	
Comorbidities				0.471
No	40	19	21	
Yes	98	40	58	
Tumor diameter(mm)				0.037
< 50	68	23	45	
≥ 50	70	36	34	
Tumor location				0.783
Upper	63	26	37	
Middle	27	12	15	
Lower	33	16	17	
Mix	15	5	10	
T stage				0.471
T1 + T2	77	35	42	
T3 + T4	61	24	37	
N stage				0.181
N0	46	16	30	
N1	92	43	49	
Surgical method				0.103
Open	43	14	29	
Laparoscopic	95	45	50	
Gastrectomy extent				0.67
Total	101	45	56	
Distal	33	13	20	
Proximal	4	1	3	
Pathological type				0.318
NET	12	3	9	
NEC	52	21	31	
MANEC	74	35	39	
Ki-67 positive index (%)				0.439
< 60	59	23	36	
≥ 60	79	36	43	
Complications				0.984
No	82	35	47	
Yes	56	24	32	
Adjuvant chemotherapy				0.193
No	66	32	34	
Yes	72	27	45	

*SMI* Skeletal muscle index, *BMI* Body mass index, *ASA* American Society of Anesthesiologists, *NET* Neuroendocrine tumor, *NEC* Neuroendocrine carcinoma, *MANEC* Mixed adenoneuroendocrine carcinoma

### Effects of sarcopenia on postoperative complications

In the present study, postoperative complications occurred in 44 patients (31.9%), and serious complications occurred in 3 patients (2.2%). The incidence of total postoperative complications was 33.9 and 30.4%, and the incidence of serious complications was 0 and 3.7% in the sarcopenia group and the nonsarcopenia group, respectively (all *p* > 0.05). Postoperative surgical and systemic complications occurred in 20 patients (14.5%) and 24 patients (17.4%), respectively, in the whole group. In the sarcopenia group and the nonsarcopenia group, the incidence of postoperative surgical complications was 13.6 and 15.2%, and the incidence of postoperative systemic complications was 20.3 and 15.2%, respectively (all *p* > 0.05). In addition, the analysis did not reveal significant differences in the incidence of specific types of complications defined according to the physical location of the complication between the two groups (all *p* > 0.05) (Table 2).

**Table 2:** Postoperative complications in 138 patients [Case(%)]

	Sarcopenia	Nonsarcopenia	P
Total complications	20 (33.9)	24 (30.4)	0.661
Serious complications	0 (0)	3 (3.7)	0.26
Surgical complications	8 (13.6)	12 (15.2)	0.788
Systemic complications	12 (20.3)	12 (15.2)	0.43
Physical location			
Pulmonary infection	12 (20.3)	12 (15.2)	0.43
Abdominal infection	4 (6.8)	3 (3.8)	0.461
Incision infection	1 (1.7)	0 (0)	0.428
Chylous fistula	0 (0)	3 (3.8)	0.26
Intestinal obstruction	0 (0)	2 (2.5)	0.507
Anastomotic fistula	1 (1.7)	2 (2.5)	1
Abdominal bleeding	1 (1.7)	2 (2.5)	1
Anastomotic stenosis	1 (1.7)	0 (0)	0.428

### Effects of sarcopenia on the prognosis of patients with g-NENs

**Fig. 1:**
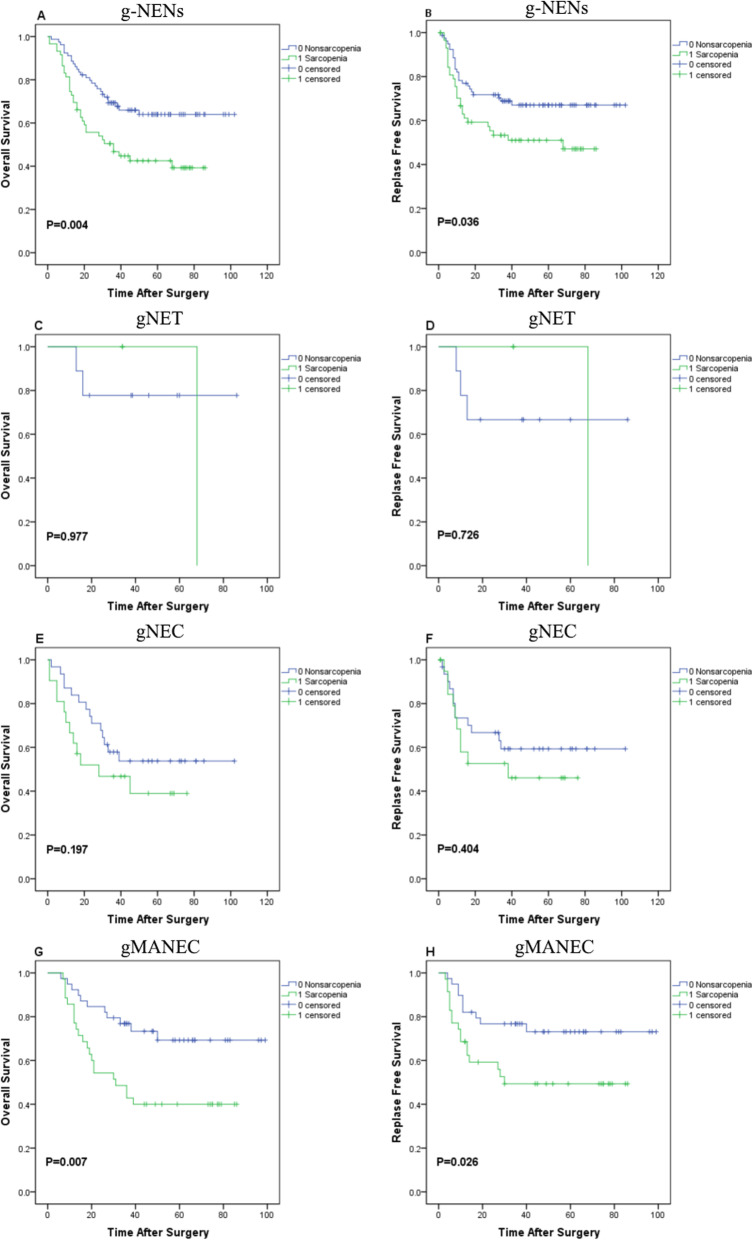
Kaplan-Meier analysis of the 3-year overall survival (OS) and recurrence-free survival (RFS) rates of patients with gastric neuroendocrine neoplasms (g-NENs) stratified according to the presence of sarcopenia (**a**-**b**) and pathological types: **c**-**d** gastric neuroendocrine tumor (gNET), **e**-**f** gastric neuroendocrine carcinoma (gNEC), and (**g**-**h**) gastric mixed adenoneuroendocrine carcinoma (gMANEC)

The 3-year OS rates were 42.37 and 65.82%, and the 3-year RFS rates were 52.54 and 68.35% in the sarcopenia and nonsarcopenia groups, respectively (all *p* < 0.05, Fig. 1a-b). According to the univariate analysis, the Anesthesiology Society of America (ASA) score, pathological T stage (pT), pathological N stage (pN), Ki-67-positive index and sarcopenia were related to the 3-year OS rates, whereas the ASA score, pN, Ki-67-positive index, and sarcopenia were related to the 3-year RFS rates (all *p* < 0.05, Table 3). The multivariate analysis only identified relations between the ASA score, pN, Ki-67-positive index and sarcopenia with the 3-year OS and RFS rates (all p < 0.05, Table 3).

**Table 3:** Uni- and multivariate analyses of factors associated with 3-year overall survival (OS) and recurrence-free survival (RFS) rates in g-NENs patients

Variable	Univariate analysis		Multivariate analysis		Univariate analysis		Multivariate analysis	
	3-year OS		3-year OS		3-year RFS		3-year RFS	
	HR (95% CI)	P	HR (95% CI)	P	HR (95% CI)	P	HR (95% CI)	P
Gender								
Male	1				1			
Female	0.650 (0.338–1.248)	0.195			0.813 (0.419–1.580)	0.542		
Age (years)								
< 65	1				1			
≥ 65	1.112 (0.669–1.847)	0.683			0.851 (0.488–1.483)	0.569		
BMI(kg/m2)								
< 25	1				1			
≥ 25	0.694 (0.330–1.460)	0.336			0.709 (0.320–1.570)	0.396		
ASA								
1	1		1		1		1	
2	1.934 (1.118–3.347)	0.018	1.869 (1.069–3.269)	0.028	2.118 (1.196–3.749)	0.010	2.191 (1.223–3.924)	0.008
3	2.54 (1.172–5.504)	0.018	2.029 (0.917–4.486)	0.081	1.294 (0.487–3.437)	0.605	0.875 (0.324–2.361)	0.791
Comorbidity								
No	1				1			
Yes	1.346 (0.751–2.411)	0.318			0.993 (0.552–1.785)	0.981		
Tumor (mm)								
< 50	1				1			
≥ 50	1.596 (0.957–2.659)	0.073			1.449 (0.841–2.496)	0.181		
Tumor location								
Upper	1				1			
Middle	0.664 (0.314–1.403)	0.283			0.778 (0.349–1.734)	0.540		
Lower	0.917 (0.484–1.735)	0.789			1.104 (0.571–2.135)	0.769		
Mix	1.253 (0.593–2.649)	0.555			1.147 (0.494–2.664)	0.749		
T stage								
T1 + T2	1		1		1			
T3 + T4	1.748 (1.054–2.898)	0.031	1.445 (0.843–2.476)	0.181	1.568 (0.914–2.691)	0.103		
N stage								
N0	1		1		1		1	
N1	5.032 (2.385–10.616)	<.001	3.554 (1.624–7.778)	0.002	5.882 (2.507–13.804)	<.001	4.710 (1.966–11.283)	0.001
Surgical method								
Open	1				1			
Laparoscopic	0.797 (0.472–1.344)	0.395			0.875 (0.496–1.546)	0.647		
Gastrectomy extent								
Total	1				1			
Distal	0.74 (0.400–1.368)	0.337			1.080 (0.584–1.994)	0.807		
Proximal	0.418 (0.058–3.029)	0.388			0.528 (0.072–3.850)	0.529		
Pathological type								
NET	1				1			
NEC	2.352 (0.712–7.773)	0.161			1.521 (0.524–4.414)	0.441		
MANEC	1.839 (0.563–6.008)	0.313			1.172 (0.410–3.350)	0.767		
Ki-67 positive index (%)								
< 60	1		1		1		1	
≥ 60	4.753 (2.469–9.152)	<.001	3.492 (1.772–6.879)	<.001	5.978 (2.810–12.718)	<.001	4.304 (1.981–9.350)	<.001
Complication								
No	1				1			
Yes	1.645 (0.994–2.723)	0.053			1.245 (0.699–2.220)	0.457		
Adjuvant chemotherapy								
No	1				1			
Yes	1.409 (0.843–2.355)	0.191			1.559 (0.894–2.719)	0.117		
Martin et al. [27]								
High	1				1			
Low	1.181 (0.709–1.968)	0.523			1.377 (0.790–2.400)	0.260		
SMI								
High	1		1		1		1	
Low	2.061 (1.243–3.420)	0.005	2.098 (1.239–3.553)	0.006	1.758 (1.025–3.018)	0.041	1.780 (1.029–3.076)	0.039

*g-NENs* gastric neuroendocrine neoplasms, *HR* Hazard ratio, *CI* Confidence interval, *BMI* Body mass index, *ASA* American Society of Anesthesiologists, *NET* Neuroendocrine tumor, *NEC* Neuroendocrine carcinoma, *MANEC* Mixed adenoneuroendocrine carcinoma, *SMI* Skeletal muscle index

### Effects of sarcopenia on the prognosis of patients with different types of g-NENs

According to the analysis stratified by postoperative pathological types, the 3-year OS rates of the sarcopenia group and nonsarcopenia group of patients with gNET were 66.67 and 77.78%, respectively, and the 3-year RFS rates were 66.67 and 66.67%, respectively (all *p* > 0.05, Fig. 1 c-d). Among patients with gNEC, the 3-year OS rates of the sarcopenia group and nonsarcopenia group were 42.86 and 54.84%, respectively, and the 3-year RFS rates were 52.38 and 61.29%, respectively (all *p* > 0.05, Fig. 1e-f). Among patients with gMANEC, the 3-year OS rates of the sarcopenia group and nonsarcopenia group were 40.00 and 71.79%, respectively, and the 3-year RFS rates were 51.43 and 74.36%, respectively (all *p* < 0.05, Fig. 1g-h). To, We subsequently performed a multivariate analysis of each subgroup of the population to more accurately evaluate the effect of sarcopenia on the prognosis of patients with different types of g-NENs. However, because few patients were included in the gNET subgroup, the Kaplan-Meier analysis did not reveal a significant difference between the two groups in the gNET subgroup. Therefore, the gNET subgroup was not included in further multivariate analyses. The multivariate analysis revealed associations between the 3-year OS rates and comorbidities, pN and the Ki-67-positive index (all *p* < 0.05), and the 3-year RFS rates were associated with pN and the Ki-67-positive index (all p < 0.05); neither OS rates nor RFS rates were associated with sarcopenia in patients with gNEC (Supplemental Table 2). However, in patients gMANEC, the pN, Ki-67-positive index and sarcopenia were related to the 3-year OS rates and the 3-year RFS rates (all p < 0.05, Table 4).

**Table 4:** Uni- and multivariate analyses of factors associated with 3-year overall survival (OS) and recurrence-free survival (RFS) rates in gMANEC patients

Variable	Univariate analysis		Multivariate analysis		Univariate analysis		Multivariate analysis	
	3-year OS		3-year OS		3-year RFS		3-year RFS	
	HR (95% CI)	P	HR (95% CI)	P	HR (95% CI)	P	HR (95% CI)	P
Gender								
Male	1				1			
Female	0.788 (0.341–1.823)	0.578			1.020 (0.431–2.412)	0.964		
Age (years)								
< 65	1				1			
≥ 65	1.234 (0.616–2.472)	0.554			0.929 (0.431–2.002)	0.851		
BMI(kg/m2)								
< 25	1				1			
≥ 25	0.856 (0.300–2.442)	0.772			0.809 (0.243–2.687)	0.729		
ASA								
1	1		1		1		1	
2	2.261 (1.038–4.929)	0.04	1.548 (0.701–3.422)	0.280	2.573 (1.141–5.801)	0.041	2.089 (0.917–4.758)	0.080
3	3.732 (1.371–10.156)	0.01	1.898 (0.628–5.730)	0.256	1.563 (0.429–5.695)	0.499	0.744 (0.197–2.813)	0.663
Comorbidity								
No	1				1			
Yes	0.846 (0.401–1.789)	0.662			0.528 (0.245–1.139)	0.104		
Tumor (mm)								
< 50	1				1			
≥ 50	1.528 (0.754–3.098)	0.239			1.264 (0.591–2.701)	0.546		
Tumor location								
Upper	1				1			
Middle	0.573 (0.188–1.741)	0.326			0.802 (0.255–2.521)	0.706		
Lower	0.852 (0.369–1.970)	0.708			1.100 (0.456–2.656)	0.832		
Mix	1.625 (0.583–4.531)	0.353			1.140 (0.317–4.094)	0.841		
T stage								
T1 + T2	1		1		1			
T3 + T4	2.197 (1.082–4.464)	0.029	2.145 (0.985–4.668)	0.055	1.753 (0.818–3.756)	0.149		
N stage								
N0	1		1		1		1	
N1	4.586 (1.756–11.979)	0.002	3.134 (1.148–8.551)	0.026	4.558 (1.568–13.249)	0.005	3.956 (1.313–11.917)	0.015
Surgical method								
Open	1				1			
Laparoscopic	0.698 (0.330–1.474)	0.346			0.778 (0.340–1.779)	0.552		
Gastrectomy extent								
Total	1				1			
Distal	0.691 (0.310–1.540)	0.366			1.201 (0.539–2.673)	0.654		
Proximal	0 (0)	0.982			0(0)	0.984		
Ki-67 positive index (%)								
< 60	1		1		1		1	
≥ 60	4.874 (1.872–12.689)	0.001	3.710(1.372–10.033)	0.010	11.553(2.729–48.913)	0.001	8.210(1.912–35.256)	0.005
Complication								
No	1				1			
Yes	1.645 (0.820–3.298)	0.161			1.200 (0.525–2.742)	0.666		
Adjuvant chemotherapy								
No	1				1			
Yes	1.428 (0.697–2.925)	0.33			1.248 (0.579–2.691)	0.572		
Martin et al. [27]								
High	1				1			
Low	1.667 (0.789–3.523)	0.181			1.868 (0.817–4.272)	0.138		
SMI								
High	1		1		1		1	
Low	2.639 (1.270–5.483)	0.009	2.735 (1.246–6.001)	0.012	2.356 (1.077–5.153)	0.032	2.825 (1.250–6.386)	0.013

*gMANEC* gastric mixed adenoneuroendocrine carcinoma, *HR* Hazard ratio, *CI* Confidence interval, *BMI* Body mass index, *ASA* American Society of Anesthesiologists, *SMI* Skeletal muscle index

## Discussion

g-NENs are a type of digestive system tumor with different clinical symptoms and biological characteristics [28]. Patients with different prognoses must be identified according to their clinical and pathological conditions to provide individualized treatment and improve the efficacy of g-NEN treatments. However, few studies have evaluated the prognostic factors for patients with g-NENs [8, 9]. Recently, the effects of preoperative body composition parameters (such as skeletal muscle mass) on postoperative short-term and long-term outcomes has attracted the attention of scholars in the East and the West. Sarcopenia is characterized by a progressive decrease in systemic muscle mass, muscle strength, or muscle physiological function associated with aging [29]. Sarcopenia has been shown to be closely related to the prognosis of patients with various malignant tumors [10–16]. However, the effect of sarcopenia on the prognosis of patients with g-NENs undergoing radical gastrectomy has not been reported. Therefore, this study combined the clinicopathological data from 138 patients treated at two institutions to explore the effects of sarcopenia on the short-term and long-term postoperative outcomes of patients with g-NENs.

Based on the definition of sarcopenia provided by the European Working Group on Sarcopenia (EWGSOP) [30] and the Asian Working Group for Sarcopenia (AWGS) [31], sarcopenia is characterized by a low skeletal muscle mass, low muscle strength and poor low physical performance. However, in the current study, low skeletal muscle mass was used as the main definition of sarcopenia. A meta-analysis exploring the relationship between sarcopenia and the risk of postoperative complications of gastrointestinal tumors included 29 studies related to sarcopenia, of which 26 used low skeletal muscle mass as the definition of sarcopenia [32]. In both studies from Eastern [10, 11] and Western [21, 27, 29] countries, researchers tend to use a low skeletal muscle mass as the definition for sarcopenia. Data on the patient’s muscle mass are obtained by analyzing the abdominal CT scan [10]. An abdominal CT scan is also a routine follow-up test performed in patients with g-NENs after radical gastrectomy [33]. The use of a low skeletal muscle mass as the definition for sarcopenia may help clinicians to make treatment decisions more conveniently and quickly.

Currently, the value of the cutoff point of sarcopenia remains controversial. The most commonly used definitions were provided by Prado et al. [21] and Martin et al. [27]. In the past, our center used X-tile software to analyze the 3-year OS rates of 924 patients with gastric adenocarcinoma after R0 resection and defined sarcopenia as a SMI < 32.5 cm^2^/m^2^ for males and a SMI < 28.6 cm^2^/m^2^ for females [10]. However, when previous definitions were applied, only the definitions reported by Martin et al. obtained a prevalence of sarcopenia similar to the values reported in previous studies (Supplementary Table 3). Therefore, we included the cutoff point defined by Martin et al. in the analysis. The Kaplan-Meier analysis and Cox regression analysis indicated that the cutoff points defined by Martin et al. were unable to serve as prognostic factors for patients with g-NENs in our study (Tables 3 and 4, Supplemental Table 2, and Supplementary Fig. 3). Therefore, this study used X-tile software to analyze the 3-year OS rates of 138 patients with g-NENs from the two institutions and defined a SMI < 44.3 cm^2^/m^2^ for males and a SMI < 32.4 cm^2^/m^2^ for females as sarcopenia, and the incidence of sarcopenia in our study was 42.8% (59/138). A significance difference in survival was not observed among the female group (Supplementary Fig. 2), perhaps because the proportion of female patients in this study was relatively small (33/138 cases, 23.9%). However, in the previous studies of sarcopenia, different values for the cutoff point of sarcopenia are usually used in male and female groups [14, 15, 27, 34], mainly because substantial differences in the strength and quality of skeletal muscle exist between males and females. In the present study, we compared the average SMI in male and female patients with g-NENs and observed a significant difference in the average value of the SMI between males and females (45.2 cm^2^/m^2^ in male, 37.5 cm^2^/m^2^ in female, *p* < 0.05). Therefore, we used different diagnostic criteria for men and women in this study to better evaluate the effect of sarcopenia on the prognosis of patients with g-NENs.

The effect of sarcopenia on short-term postoperative outcomes in patients with malignant tumors remains controversial. Previous studies have confirmed that sarcopenia is associated with the postoperative short-term prognosis in patients with multiple malignant tumors [11, 13, 15, 35]. In a Chinese study, an analysis of 937 patients with gastric cancer after radical gastrectomy showed that sarcopenia was related to severe postoperative complications [11]. An American study identified an association between sarcopenia and the short-term outcomes in patients with pancreatic cancer after pancreatectomy [35]. However, some studies have reported the opposite results [34, 36]. As shown in the study by Tegels [34], the incidence of sarcopenia is higher in patients with gastric cancer, but it is not associated with a poor postoperative prognosis. According to Ouchi [36], sarcopenia does not increase the incidence of total and severe postoperative complications in patients with colorectal cancer [36]. In the present study, significant differences in the incidences of total postoperative complications, surgical complications and systemic complications were not observed between the patients with g-NENs presenting with and without sarcopenia. After stratification according to the physical location of the complications, significant correlations were not observed between sarcopenia and specific types of complications in patients with g-NENs.

In recent years, studies have confirmed that sarcopenia is closely related to the long-term prognoses of patients with multiple malignant tumors [10, 12, 14, 16]. Studies by Voron have identified sarcopenia as an independent prognostic factor for long-term outcomes in patients with hepatocellular carcinoma after hepatectomy [12]. As shown in the study by Tan, sarcopenia is associated with a poor prognosis for patients with pancreatic cancer [16]. Similar to previous studies, preoperative sarcopenia was an independent risk factor for the long-term prognosis of patients with g-NENs in the present study. We also examined the interactions between sarcopenia and the gastrectomy status and tumor aggressiveness. No significant differences in surgical methods, the extent of laparoscopic gastrectomy and pathological stages were observed between the sarcopenia group and the nonsarcopenia group (Table 1). The multivariate analysis identified the pN stage and sarcopenia as independent prognostic factors for 3-year OS and RFS rates in patients with g-NENs, while surgical methods, the extent of laparoscopic gastrectomy and pT stage were not associated with survival (Table 3). The HR value of sarcopenia changed little between the univariate and multivariate analyses in our study (Table 3). Thus, the prognostic effect of preoperative sarcopenia is less affected by the gastrectomy status and tumor aggressiveness in patients with g-NENs. However, g-NENs are divided into three different pathological types, namely, gNET, gNEC, and gMANEC. The degree of tumor differentiation, grade, and cellular components of the three pathological types are not the same [4], and the treatment strategy and prognosis are also significantly different in patients with different pathological types [37]. In the present study, a further stratified analysis showed relations between sarcopenia and the 3-year OS and RFS rates in patients with gMANEC. Potential explanations for this result are provided below. First, for the subgroup of the gNET population, gNET is a highly differentiated neuroendocrine tumor, with mainly low or moderate malignancy, and presents as stage G1 and G2 [3]. The lower tumor invasiveness and the lower effect on skeletal muscle mass may explain why sarcopenia is not useful as a prognostic factor for patients with gNET. This result also may caused by the relatively small number of gNET patients, further study may be required. Second, compared with gNEC and gMANEC, gNEC is a poorly differentiated neuroendocrine carcinoma, which is generally highly malignant and manifests as stage G3. gMANEC is defined as a malignant tumor with morphological components of glandular epithelial cells and neuroendocrine cells, both of which account for at least more than 30% of the total cells [4]. The clinical characteristics of gMANEC generally depend on the proportion of neuroendocrine carcinoma components [38, 39]. Fernandes et al. postulated that the prognosis of patients with gMANEC might be related to whether certain tumor components are more invasive [40]. Furthermore, previous studies have confirmed that sarcopenia is associated with the long-term prognosis of patients with gastric adenocarcinoma [10, 11]. Therefore, we propose that the mechanism may be modulated by the presence of more adenocarcinoma components in gMANEC, and thus, sarcopenia is only related to the long-term prognosis of patients with gMANEC, but not the patients with gNET and gNEC, in the present study. The underlying molecular mechanism must be further elucidated. This result may be caused by the sample sizes of individual subgroups. Further study with bigger sample sizes of different pathological types needed to be conducted.

This study had some limitations. First, because most patients with gNET received endoscopic treatment, the number of patients with gNET included in this study was limited, which may cause bias. Second, this study employed a retrospective case-control design and was conducted in an Asian population; therefore, the results must be confirmed by prospective studies and data from Western countries. Third, the proportion of female patients in this study is relatively small (33/138 cases, 23.9%), and thus the prognostic effect of sarcopenia on female patients with g-NENs must be further analyzed in a study with a larger population. We plan to conduct related studies in the future. Fourth, this study did not analyze the effects of postoperative adjuvant chemotherapy and postoperative sarcopenia caused by the gastrectomy status and tumor aggressiveness on long-term outcomes, which may also bias the results. Fifth, Due to the relatively few cases of stratified analysis of pathological subtypes, we did not identify sarcopenia scores related to tumour type gNET, gNEC and gMANEC, and the prognostic value of sarcopenia for g-NENs may be biased. In the future, a larger sample size is needed to determine the best cut-off point of sarcopenia with different pathological types, and to verify the prognostic effect of sarcopenia on different pathological types of g-NENs. Nevertheless, to our knowledge, this study is the first to explore the effects of sarcopenia on the short-term and long-term outcomes in patients with g-NENs by using data from two independent large-volume institutions, thus providing a reference for future clinical trials.

## Conclusions

In the present study, a SMI < 44.3 cm^2^/m^2^ for males and a SMI < 32.4 cm^2^/m^2^ for females were identified as the optimal cutoff points for sarcopenia in patients with g-NENs. Sarcopenia was not significantly associated with postoperative complications in patients with g-NENs. Sarcopenia is an independent risk factor for the long-term prognosis of patients with gMANEC undergoing radical gastrectomy. Further multicenter prospective studies are needed to confirm the prognostic value of sarcopenia in patients with g-NENs.

## Supplementary information

**Additional file 1 **: **Supplemental Table 1.** Clinicopathological characteristics of patients treated at FMUUH and FMUFAH.

**Additional file 2 **: **Supplemental Table 2.** Univariate and multivariate analyses of factors associated with 3-year overall survival (OS) and recurrence-free survival (RFS) rates in patients with gNEC.

**Additional file 3 **: **Supplemental Table 3.** Four cutoff points tested as thresholds to define sarcopenia and the prevalence of sarcopenia.

**Additional file 4 **: **Supplemental Figure 1**. Computed tomography (CT) image captured at the third lumbar vertebral (L3) level. The following skeletal muscles are outlined in red: rectus abdominis; psoas, quadratus lumborum, paraspinal, transverse abdominal, external oblique, internal oblique, and rectus abdominis muscles. This male patient with sarcopenia had an L3 muscle index of 52.35 cm^2^/m^2^.

**Additional file 5 **: **Supplemental Figure 2.** The cutoff points of the skeletal muscle index (SMI) for sarcopenia defined by X-tile software. (A) X-tile plots for males (44.3 cm^2^/m^2^, χ^2^ = 4.2611, *p* = 0.038) and (B) females (32.4 cm^2^/m^2^, χ^2^ = 1.0039, *p* = 0.214) are shown.

**Additional file 6 **: **Supplemental Figure 3.** Kaplan-Meier analysis of the 3-year overall survival (OS) and recurrence-free survival (RFS) rates of patients with gastric neuroendocrine neoplasms (g-NENs) stratified according to the presence of sarcopenia diagnosed by the skeletal muscle index (SMI) cutoff points (for male, 43.0 cm2/m2 for BMI < 25 kg/m2, 53.0 cm2/m2 for BMI ≥25 kg/m2; for female, 41 cm2/m2) defined by Martin et al. (A-B) and pathological types: (C-D) gastric neuroendocrine tumor (gNET), (E-F) gastric neuroendocrine carcinoma (gNEC), (G-H) gastric mixed adenoneuroendocrine carcinoma (gMANEC).

## Data Availability

The datasets used and/or analyzed during the current study are available from the corresponding author on reasonable request.

## Abbreviations

g-NENs: gastric neuroendocrine neoplasms
OS: Overall survival
RFS: Recurrence-free survival
SMI: Skeletal muscle index
BMI: Body mass index
ASA: American Society of Anesthesiologists
gNET: gastric neuroendocrine tumor
gNEC: gastric neuroendocrine carcinoma
gMANEC: gastric mixed adenoneuroendocrine carcinoma
