# Supplementary material for: Effect of sarcopenia on short- and long-term outcomes in patients with gastric neuroendocrine neoplasms after radical gastrectomy: results from a large, two-institution series
Source: BMC Cancer. 2020 Oct 15;20:1002. doi: 10.1186/s12885-020-07506-9 (PMC7560019; doi:10.1186/s12885-020-07506-9)
Supplement: Supplementary file 1 — Additional file 1 : Supplemental Table 1. Clinicopathological characteristics of patients treated at FMUUH and FMUFAH. [file 12885_2020_7506_MOESM1_ESM.docx]

| **SUPPLEMENTARY TABLE 1 Clinicopathological characteristics in FMUUH and FMUFAH.** | | | | |
| --- | --- | --- | --- | --- |
| Variable | All(n=138) | FMUUH(n=111) | FMUFAH(n=27) | P |
| Gender |  |  |  | 0.785 |
| Male | 105 | 85 | 20 |  |
| Female | 33 | 26 | 7 |  |
| Age(years) |  |  |  | 0.006 |
| <65 | 80 | 58 | 22 |  |
| ≥65 | 58 | 53 | 5 |  |
| BMI(kg/m2 ) |  |  |  | 0.158 |
| <25 | 115 | 95 | 20 |  |
| ≥25 | 23 | 16 | 7 |  |
| ASA |  |  |  | 0.812 |
| 1 | 69 | 55 | 14 |  |
| 2 | 54 | 43 | 11 |  |
| 3 | 15 | 13 | 2 |  |
| Comorbidities |  |  |  | 0.048 |
| No | 40 | 28 | 12 |  |
| Yes | 98 | 83 | 15 |  |
| Tumor diameter(mm) |  |  |  | 0.896 |
| <50 | 68 | 55 | 13 |  |
| ≥50 | 70 | 56 | 14 |  |
| Tumor location |  |  |  | 0.678 |
| Upper | 63 | 50 | 13 |  |
| Middle | 27 | 20 | 7 |  |
| Lower | 33 | 28 | 5 |  |
| Mix | 15 | 13 | 2 |  |
| T stage |  |  |  | 0.001 |
| T1+T2 | 77 | 70 | 7 |  |
| T3+T4 | 61 | 41 | 20 |  |
| N stage |  |  |  | 0.172 |
| N0 | 46 | 40 | 6 |  |
| N1 | 92 | 71 | 21 |  |
| Surgical method |  |  |  | <.001 |
| Open | 43 | 21 | 22 |  |
| Laparoscopic | 95 | 90 | 5 |  |
| Gastrectomy extent |  |  |  | 0.297 |
| Total | 101 | 82 | 19 |  |
| Distal | 33 | 27 | 6 |  |
| Proximal | 4 | 2 | 2 |  |
| Pathological type |  |  |  | <.001 |
| NET | 12 | 11 | 1 |  |
| NEC | 52 | 32 | 20 |  |
| MANEC | 74 | 68 | 6 |  |
| Ki-67 positive index (%) |  |  |  | 0.27 |
| <60 | 59 | 50 | 9 |  |
| ≥60 | 79 | 61 | 18 |  |
| Complications |  |  |  | 0.648 |
| No | 82 | 67 | 15 |  |
| Yes | 56 | 44 | 12 |  |
| Adjuvant chemotherapy |  |  |  | 0.211 |
| No | 66 | 56 | 10 |  |
| Yes | 72 | 55 | 17 |  |
| SMI |  |  |  | 0.005 |
| High | 79 | 57 | 22 |  |
| Low | 59 | 54 | 5 |  |
| FMUUH, Fujian Medical University Union Hospital;FMUFAH, First Affiliated Hospital of Fujian Medical University;BMI, body mass index;ASA, American Society of Anesthesiologists;NET, neuroendocrine tumor;NEC, neuroendocrine carcinoma;MANEC, mixed adenoneuroendocrine carcinoma;SMI, skeletal muscle index. | | | | |
