# Supplementary material for: Effect of sarcopenia on short- and long-term outcomes in patients with gastric neuroendocrine neoplasms after radical gastrectomy: results from a large, two-institution series
Source: BMC Cancer. 2020 Oct 15;20:1002. doi: 10.1186/s12885-020-07506-9 (PMC7560019; doi:10.1186/s12885-020-07506-9)
Supplement: Supplementary file 2 — Additional file 2 : Supplemental Table 2. Univariate and multivariate analyses of factors associated with 3-year overall survival (OS) and recurrence-free survival (RFS) rates in patients with gNEC. [file 12885_2020_7506_MOESM2_ESM.docx]

**SUPPLEMENTARY TABLE 2 Uni- and multivariate analyses of factors associated with 3-year overall survival (OS) and recurrence-free survival (RFS) rates in gNEC patients.**

| Variable | Univariate analysis | | Multivariate analysis | | Univariate analysis | | Multivariate analysis | |
| --- | --- | --- | --- | --- | --- | --- | --- | --- |
|  | 3-year OS | | 3-year OS | | 3-year RFS | | 3-year RFS | |
|  | HR (95% CI) | P | HR (95% CI) | P | HR (95% CI) | P | HR (95% CI) | P |
| Gender |  |  |  |  |  |  |  |  |
| Male | 1 |  |  |  | 1 |  |  |  |
| Female | 0.465 (0.139-1.551) | 0.213 |  |  | 0.370 (0.087-1.586) | 0.181 |  |  |
| Age(years) |  |  |  |  |  |  |  |  |
| <65 | 1 |  |  |  | 1 |  |  |  |
| ≥65 | 0.836 (0.379-1.844) | 0.658 |  |  | 0.704 (0.295-1.678) | 0.428 |  |  |
| BMI(kg/m2 ) |  |  |  |  |  |  |  |  |
| <25 | 1 |  |  |  | 1 |  |  |  |
| ≥25 | 0.723 (0.249-2.102) | 0.552 |  |  | 0.908 (0.307-2.683) | 0.861 |  |  |
| ASA |  |  |  |  |  |  |  |  |
| 1 | 1 |  |  |  | 1 |  |  |  |
| 2 | 1.893 (0.817-4.385) | 0.137 |  |  | 2.307 (0.917-5.805) | 0.076 |  |  |
| 3 | 2.118 (0.569-7.879) | 0.263 |  |  | 1.547 (0.321-7.467) | 0.587 |  |  |
| Comorbidity |  |  |  |  |  |  |  |  |
| No | 1 |  | 1 |  | 1 |  |  |  |
| Yes | 2.802 (1.054-7.451) | 0.039 | 2.949 (1.089-7.987) | 0.033 | 2.330 (0.858-6.327) | 0.097 |  |  |
| Tumor (mm) |  |  |  |  |  |  |  |  |
| <50 | 1 |  |  |  | 1 |  |  |  |
| ≥50 | 1.507 (0.682-3.328) | 0.31 |  |  | 1.681 (0.705-4.011) | 0.241 |  |  |
| Tumor location |  |  |  |  |  |  |  |  |
| Upper | 1 |  |  |  | 1 |  |  |  |
| Middle | 0.912 (0.300-2.772) | 0.871 |  |  | 0.871 (0.245-3.087) | 0.830 |  |  |
| Lower | 1.899 (0.677-5.322) | 0.223 |  |  | 1.451 (0.465-4.530) | 0.522 |  |  |
| Mix | 1.036 (0.297-3.608) | 0.956 |  |  | 1.296 (0.337-4.239) | 0.782 |  |  |
| T stage |  |  |  |  |  |  |  |  |
| T1+T2 | 1 |  |  |  | 1 |  |  |  |
| T3+T4 | 1.175 (0.544-2.538) | 0.681 |  |  | 1.415 (0.611-3.276) | 0.418 |  |  |
| N stage |  |  |  |  |  |  |  |  |
| N0 | 1 |  | 1 |  | 1 |  | 1 |  |
| N1 | 5.718 (1.344-24.328) | 0.018 | 5.759(1.332-24.896) | 0.019 | 9.578 (1.283-71.511) | 0.028 | 8.040 (1.071-60.358) | 0.043 |
| Surgical method |  |  |  |  |  |  |  |  |
| Open | 1 |  |  |  | 1 |  |  |  |
| Laparoscopic | 0.966 (0.447-2.092) | 0.931 |  |  | 0.953 (0.412-2.206) | 0.910 |  |  |
| Gastrectomy extent |  |  |  |  |  |  |  |  |
| Total | 1 |  |  |  | 1 |  |  |  |
| Distal | 1.001 (0.375-2.670) | 0.998 |  |  | 0.874 (0.294-2.598) | 0.808 |  |  |
| Proximal | 0.531 (0.071-3.963) | 0.537 |  |  | 0.593 (0.079-4.462) | 0.612 |  |  |
| Ki-67 positive index (%) |  |  |  |  |  |  |  |  |
| <60 | 1 |  | 1 |  | 1 |  | 1 |  |
| ≥60 | 5.713 (1.708-19.112) | 0.005 | 4.360(1.280-14.854) | 0.019 | 7.267 (1.691-31.243) | 0.008 | 6.318 (1.463-27.279) | 0.014 |
| Complication |  |  |  |  |  |  |  |  |
| No | 1 |  |  |  | 1 |  |  |  |
| Yes | 1.287 (0.595-2.786) | 0.522 |  |  | 0.962 (0.403-2.296) | 0.931 |  |  |
| Adjuvant chemotherapy |  |  |  |  |  |  |  |  |
| No | 1 |  |  |  | 1 |  |  |  |
| Yes | 1.039 (0.480-2.249) | 0.922 |  |  | 1.297 (0.554-3.036) | 0.549 |  |  |
| Martin et al.[29] |  |  |  |  |  |  |  |  |
| High | 1 |  |  |  | 1 |  |  |  |
| Low | 0.919 (0.426-1.982） | 0.829 |  |  | 1.108 (0.479-2.566） | 0.810 |  |  |
| SMI |  |  |  |  |  |  |  |  |
| High | 1 |  |  |  | 1 |  |  |  |
| Low | 1.652 (0.762-3.582) | 0.203 |  |  | 1.420 (0.613-3.292) | 0.413 |  |  |

gNEC, gastric neuroendocrine carcinoma;HR, hazard ratio; CI confidence interval; BMI, body mass index;ASA, American Society of Anesthesiologists; SMI, skeletal muscle index.
