# Supplementary material for: Effect of sarcopenia on short- and long-term outcomes in patients with gastric neuroendocrine neoplasms after radical gastrectomy: results from a large, two-institution series
Source: BMC Cancer. 2020 Oct 15;20:1002. doi: 10.1186/s12885-020-07506-9 (PMC7560019; doi:10.1186/s12885-020-07506-9)
Supplement: Supplementary file 3 — Additional file 3 : Supplemental Table 3. Four cutoff points tested as thresholds to define sarcopenia and the prevalence of sarcopenia. [file 12885_2020_7506_MOESM3_ESM.docx]

| **SUPPLEMENTARY TABLE 3 Four cutoff points tested as a threshold to define sarcopenia and the prevalence of sarcopenia.** | | | | |
| --- | --- | --- | --- | --- |
|  |  |  |  |  |
| Study | Cutoff points for SMI to define sarcopenia | | SMI (Total n=138) | |
|  | Male | Female | High | Low |
| Martin et al.[29](Canada) | 53.0 for BMI ≥25 | 41 | 60 (43.5) | 78 (56.5) |
|  | 43.0 for BMI < 25 |  |  |  |
| Prado et al.[21]（Canada） | 52.4 | 38.5 | 28 (20.2) | 110 (79.8) |
| ZF Zheng et al.[10](China) | 32.5 | 28.6 | 131(94.9) | 7 (5.1) |
| Our Study | 44.3 | 32.6 | 79 (57.2) | 59 (42.8) |
| SMI, skeletal muscle index (cm^2^/m^2^);BMI, body mass index (kg^2^/m^2^). | | | | |

1. Martin L, Birdsell L, Macdonald N, Reiman T, Clandinin MT, McCargar LJ, et al. Cancer cachexia in the age of obesity: skeletal muscle depletion is a powerful prognostic factor, independent of body mass index. Journal of clinical oncology : official journal of the American Society of Clinical Oncology. 2013;31(12):1539-47. doi: 10.1200/jco.2012.45.2722. PubMed PMID: 23530101.

2. Prado CM, Lieffers JR, McCargar LJ, Reiman T, Sawyer MB, Martin L, et al. Prevalence and clinical implications of sarcopenic obesity in patients with solid tumours of the respiratory and gastrointestinal tracts: a population-based study. The Lancet Oncology. 2008;9(7):629-35. doi: 10.1016/s1470-2045(08)70153-0. PubMed PMID: 18539529.

3. Zheng ZF, Lu J, Zheng CH, Li P, Xie JW, Wang JB, et al. A Novel Prognostic Scoring System Based on Preoperative Sarcopenia Predicts the Long-Term Outcome for Patients After R0 Resection for Gastric Cancer: Experiences of a High-Volume Center. Annals of surgical oncology. 2017;24(7):1795-803. doi: 10.1245/s10434-017-5813-7. PubMed PMID: 28213789.
