# Supplementary figures and images for: Effect of sarcopenia on short- and long-term outcomes in patients with gastric neuroendocrine neoplasms after radical gastrectomy: results from a large, two-institution series
Source: BMC Cancer. 2020 Oct 15;20:1002. doi: 10.1186/s12885-020-07506-9 (PMC7560019; doi:10.1186/s12885-020-07506-9)

**Supplemental Figure 1**


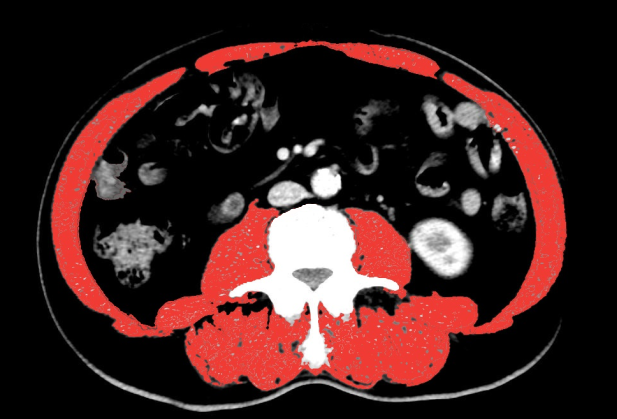

Supplement: Supplementary file 4 — Additional file 4 : Supplemental Figure 1. Computed tomography (CT) image captured at the third lumbar vertebral (L3) level. The following skeletal muscles are outlined in red: rectus abdominis; psoas, quadratus lumborum, paraspinal, transverse abdominal, external oblique, internal oblique, and rectus abdominis muscles. This male patient with sarcopenia had an L3 muscle index of 52.35 cm2/m2. [file 12885_2020_7506_MOESM4_ESM.docx]

**Supplemental Figure 2**

**
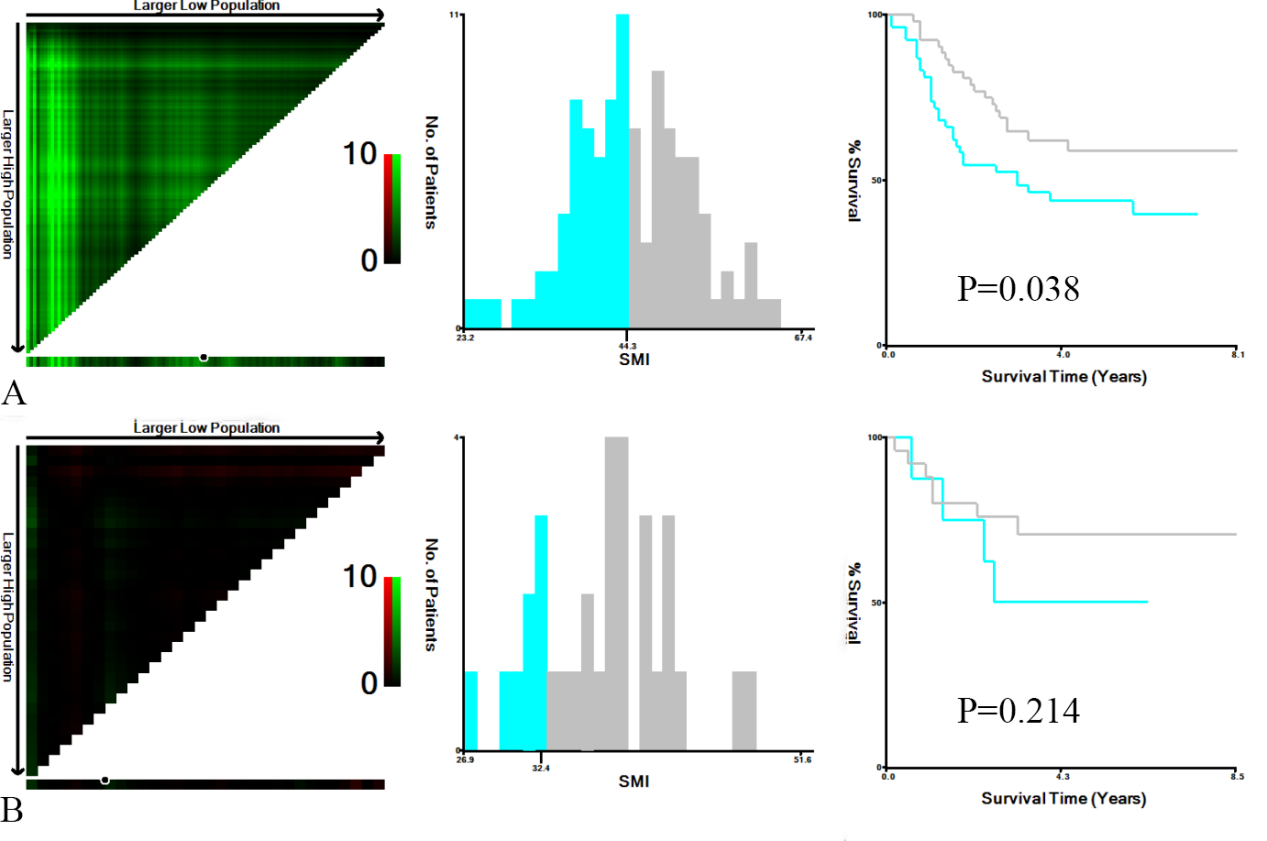
**

Supplement: Supplementary file 5 — Additional file 5 : Supplemental Figure 2. The cutoff points of the skeletal muscle index (SMI) for sarcopenia defined by X-tile software. (A) X-tile plots for males (44.3 cm2/m2, χ2 = 4.2611, p = 0.038) and (B) females (32.4 cm2/m2, χ2 = 1.0039, p = 0.214) are shown. [file 12885_2020_7506_MOESM5_ESM.docx]

**Supplemental Figure 3
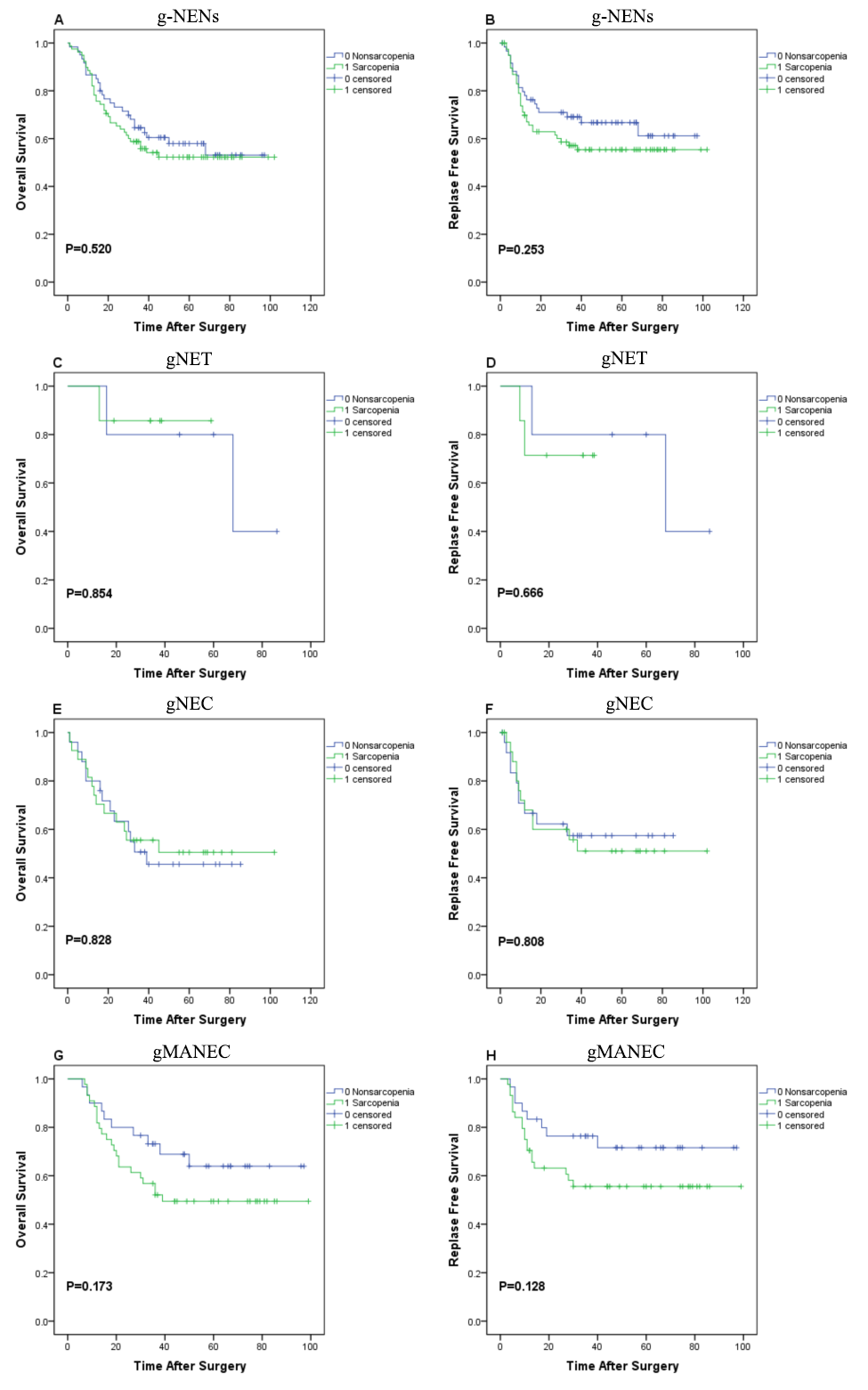
**

Supplement: Supplementary file 6 — Additional file 6 : Supplemental Figure 3. Kaplan-Meier analysis of the 3-year overall survival (OS) and recurrence-free survival (RFS) rates of patients with gastric neuroendocrine neoplasms (g-NENs) stratified according to the presence of sarcopenia diagnosed by the skeletal muscle index (SMI) cutoff points (for male, 43.0 cm2/m2 for BMI < 25 kg/m2, 53.0 cm2/m2 for BMI ≥25 kg/m2; for female, 41 cm2/m2) defined by Martin et al. (A-B) and pathological types: (C-D) gastric neuroendocrine tumor (gNET), (E-F) gastric neuroendocrine carcinoma (gNEC), (G-H) gastric mixed adenoneuroendocrine carcinoma (gMANEC). [file 12885_2020_7506_MOESM6_ESM.docx]
